# Symplectic Method Analysis of the Thermal Buckling Behavior of Graphene Origami-Reinforced Composite Beams

**DOI:** 10.3390/nano16160997

**Published:** 2026-08-13

**Authors:** Zuoquan Zhu, Mengxin Zhao, Nan Zhao, Yuyan Zhou, Haixia Du

**Affiliations:** School of Mathematics and Statistics, Zhengzhou Normal University, Zhengzhou 450044, China; zuoquanzhu@zznu.edu.cn (Z.Z.);

**Keywords:** graphene origami, thermal buckling, symplectic method, analytical solution, folding degree

## Abstract

This study constructs a buckling analysis model integrating Euler–Bernoulli beam theory and Hamiltonian formulation to clarify the buckling characteristics of graphene origami (GOri)-reinforced beams and systematically explore the structural stability of graded composite beams. Under the symplectic space framework, the thermal buckling issue of GOri composite beams is converted into a zero-eigenvalue problem, where critical thermal buckling loads and corresponding buckling modes correspond to the symplectic eigenvalues and eigenfunctions of the Hamiltonian system. Taking the through-thickness continuity of GOri fillers into consideration, analytical expressions of buckling modes and critical buckling loads are derived using bifurcation criteria and normalization operations. Afterwards, parametric investigations are conducted to reveal how GOri content, spatial distribution, ambient temperature and folding degree affect beam buckling responses. Numerical results demonstrate that GOri distribution exerts a dominant influence on the structural buckling performance; critical thermal buckling loads tend to decline with rising folding degree and temperature. Reasonable optimization of GOri layout can significantly strengthen the mechanical capacity of composite beams, which lays solid theoretical guidance for their structural design and mechanical property enhancement.

## 1. Introduction

Against the backdrop of high-end equipment evolving toward high-temperature resistance, lightweight design, and high reliability, structural buckling has become a core bottleneck limiting the operational safety of critical components in fields such as aerospace and nuclear power [1,2,3]. As the most fundamental load-bearing unit in engineering, the buckling characteristics of beam structures directly affect the mechanical performance of the overall structure. Owing to their low specific strength and limited buckling resistance, traditional metal beams and homogeneous composite beams struggle to meet the demands of extreme operating conditions, creating an urgent need to develop new types of reinforcing materials and structural systems.

Graphene origami (GOri) metamaterials combine the inherent advantages of graphene with the configurational effects of origami structures, offering adjustable stiffness and thermal deformation capabilities while maintaining a lightweight design, thereby providing a novel technical approach for enhancing the thermal buckling performance of beam structures [4,5,6]. However, such reinforced beams exhibit complex multiscale mechanical behavior, including material inhomogeneity and negative Poisson’s ratio effects; further research is urgently needed on their buckling mechanisms and solution methods. Furthermore, the analysis of beam structure buckling currently relies primarily on classical theoretical methods and numerical simulations. For example, the classical Euler–Bernoulli beam theory simplifies the buckling problem to the solution of a fourth-order differential equation by assuming a flat cross-section [7,8], which is then solved using numerical simulation or analytical methods. In terms of numerical simulation, the finite element method achieves approximate solutions by discretizing the structure, but it faces issues such as strong mesh dependency and insufficient accuracy in capturing buckling critical points, and it struggles to reveal the intrinsic relationship between microscopic parameters and macroscopic buckling behavior [9,10,11,12]; although the boundary element method reduces the dimensionality, it encounters integration singularity problems when handling material inhomogeneity [13,14,15]. Numerical methods such as the differential quadrature method [16,17,18,19], the differential transformation method [20,21], and the quadratic perturbation method [22,23,24,25] all rely on the number of iterations. Moreover, the tolerance modelling method can effectively capture the influence of microstructure size on the mechanical behaviors of composite structures, including free vibration [26,27,28] and dynamic stability [29,30]. Consequently, existing methods struggle to meet the dual requirements of accuracy and mechanistic understanding in the buckling analysis of graphene origami metamaterial-reinforced beams.

The symplectic solution method is based on the Hamiltonian mechanics framework. By introducing dual variables, it transforms high-order differential equations into a system of first-order regular equations in symplectic space, thereby providing an analytical solution approach for complex structural mechanics problems [31,32,33,34,35]. Its core advantages are as follows: First, through the symplectic orthogonality relation, it naturally satisfies complex boundary conditions such as elastic constraints and mixed boundaries, thereby avoiding the boundary transformation errors inherent in traditional methods; second, the use of piecewise symplectic transformations allows for precise handling of the transfer of mechanical quantities in structures with nonuniform cross-sections; third, it enables the direct determination of symplectic eigenvalues and eigenfunctions, revealing the essential characteristics of structural buckling. Existing research indicates that the symplectic method has made some progress in problems such as the bending of functionally graded beams and the vibration of laminated plates; however, its application to the multiscale coupled problem of buckling in graphene origami-reinforced metamaterial beams still requires further exploration. More importantly, most existing studies concentrate on the compressive buckling of graphene origami-reinforced metamaterials considering temperature dependence, whereas reports on thermally induced buckling are still limited. Therefore, the present work investigates thermally generated buckling behavior within the framework of symplectic elasticity.

In this paper, a theoretical analysis framework based on the symplectic solver method for the buckling problem of graphene origami metamaterial-reinforced beams is proposed. First, the negative Poisson’s ratio effect of graphene origami metamaterials is predicted by combining micromechanical theory with the conversion of microscopic folding parameters into macroscopic equivalent material parameters. Second, the symplectic eigenvalues and eigen-solutions under thermal loading are derived on the basis of Hamilton’s principle, and a piecewise symplectic solver model is constructed. Finally, the buckling critical load and modes are obtained through symplectic eigenvalue analysis, and a parametric analysis is conducted. The research findings not only provide an efficient and accurate solution method for the buckling analysis of novel reinforced beams but also expand the scope of application of symplectic elastic mechanics in multiscale functional material structures.

## 2. Graphene Origami Metamaterial Composite Beams

**Figure 1:**
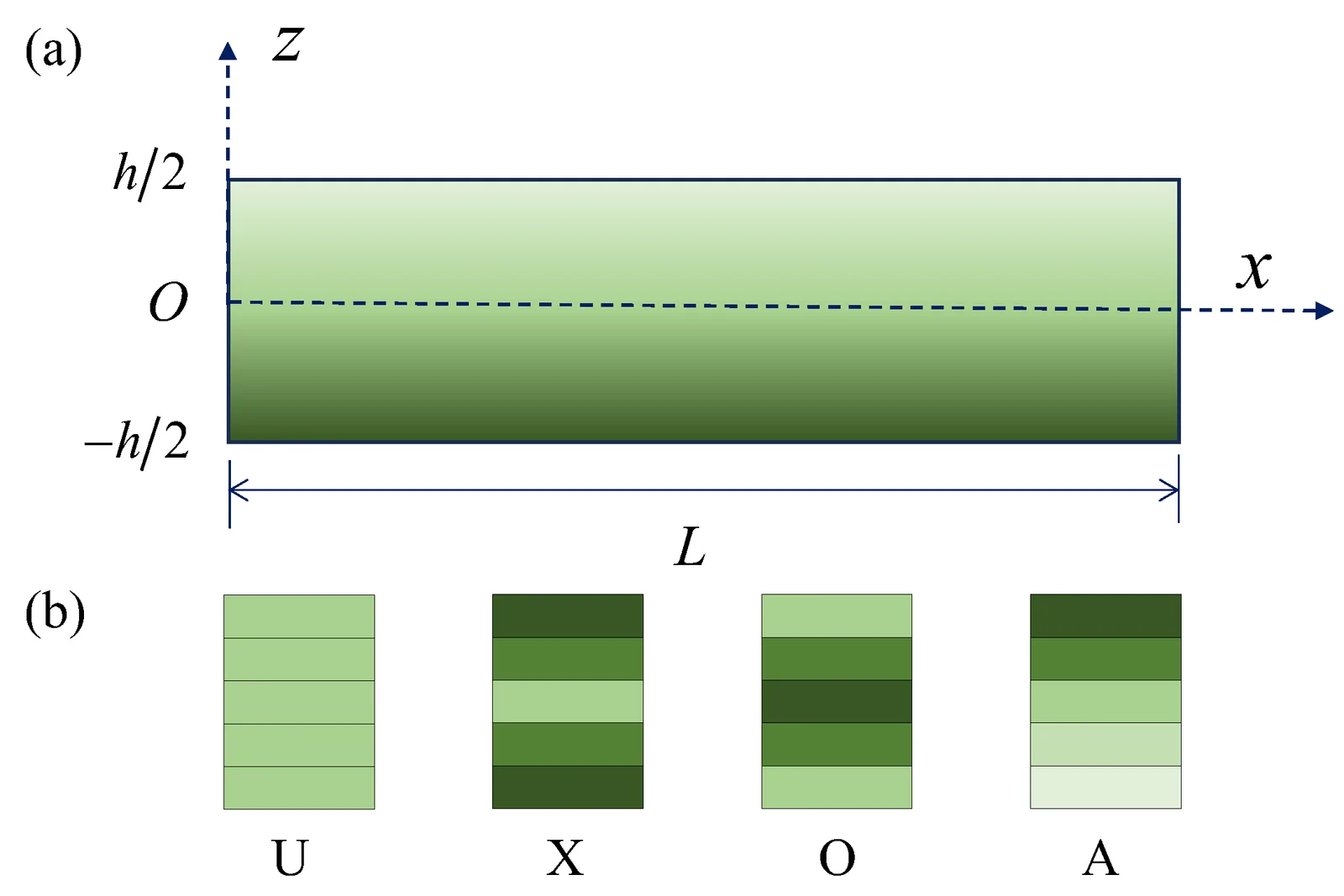
(**a**) Geometric structure of graphene origami-reinforced composite beams; (**b**) four typical forms of the graphene origami distribution.

A functionally graded GOri/Cu composite beam with length L and thickness h is considered, as shown in Figure 1a. This composite beam consists of a Cu matrix combined with a graphene origami structure and is defined using the coordinate system $\left(x,z\right)$. The origin of this coordinate system lies on the mid-plane of the shell, where the x-axis extends along the axial direction and the z-axis is perpendicular to the mid-plane (with a range of −h/2≤z≤h/2). It is assumed that the beam material is isotropic and linearly elastic, with its material properties distributed as a gradient along the thickness direction, as shown in Figure 1b. In the U-WGr configuration, graphene origami (GOri) is uniformly distributed along the thickness direction. For the O-WGr configuration, the content of GOri reaches its maximum at the geometric midsurface of the beam and gradually decreases toward the upper and lower surfaces. For the X-WGr configuration, the GOri content is the lowest at the geometric midsurface of the beam and increases gradually toward the upper and lower surfaces. In the A-WGr configuration, the GOri content is highest at one surface and decreases gradually toward the opposite surface. The degree of folding $H_{GR}$ of the graphene origami structure is determined by the degree of hydrogen atom coverage (i.e., the degree of hydrogenation). Four types of graphene origami distributions are considered, corresponding to different graphene distribution patterns spanning a single layer, with volume fractions $V_{Gr}^{\left(k\right)}$ ranging from *k* = 1 to $N_{L}$ (where $N_{L}$ is the total number of layers in the origami structure), defined as follows:

(1) $${\text{U-W}}_{\mathrm{GR}}:V_{GR}^{\left(k\right)}=V_{GR}\;{\text{X-W}}_{\mathrm{GR}}:V_{GR}^{\left(k\right)}=2V_{GR}\frac{\left|2k-N_{L}-1\right|}{N_{L}}\;{\text{O-W}}_{\mathrm{GR}}:\;V_{GR}^{\left(k\right)}=2V_{GR}\left(1-\frac{\left|2k-N_{L}-1\right|}{N_{L}}\right)\;{\text{A-W}}_{\mathrm{GR}}:{\;V}_{GR}^{\left(k\right)}=\frac{V_{GR}\left(2k-1\right)}{N_{L}}$$

where $V_{GR}$ is the total volume fraction of GOri, which can be obtained using the formula of mass fraction $W_{GR}$:

(2) $$V_{GR}=\frac{W_{\mathrm{GR}}}{W_{\mathrm{GR}}+\left(\rho_{\mathrm{GR}}/\rho_{\mathrm{Cu}}\right)\left(1-W_{\mathrm{GR}}\right)}$$

In Equation (2), $W_{\mathrm{GR}}$ is the total weight fraction of GOri, $\rho_{\mathrm{GR}}$ is the density of graphene and $\rho_{\mathrm{Cu}}$ is the density of Cu. Notably, the volume fractions of both GOri and the Cu matrix are linked to one another as follows:

(3) $$V_{\mathrm{Cu}}=1-V_{GR}$$

The equivalent elastic modulus ($E_{e}$), Poisson’s ratio ($v_{e}$), and thermal expansion coefficient ($\alpha_{e}$) of the composite material in terms of mass fraction and density are determined by the micromechanical model assisted by genetic programming (GP) proposed by Zhao et al. [36,37]:

(4) $$\begin{array}{l} E_{e}=\frac{1+\xi \lambda V_{GR}}{1-\lambda V_{GR}}E_{Cu}\times f_{E}\left(H_{GR},V_{GR},T\right)\\ v_{e}=\left(v_{GR}V_{GR}+v_{Cu}V_{Cu}\right)\times f_{v}\left(H_{GR},V_{GR},T\right)\\ \alpha_{e}=\left(\rho_{GR}V_{GR}+\rho_{Cu}V_{Cu}\right)\times f_{\alpha }\left(V_{GR},T\right) \end{array}$$

The material coefficient (λ) and size coefficient (ξ) are as follows:

(5) $$\lambda =\frac{\left(E_{GR}/E_{Cu}\right)-1}{\left(E_{GR}/E_{Cu}\right)+\xi },\xi =2\left(l_{GR}/t_{GR}\right)$$

where $l_{GR}$ and $t_{GR}$ denote the length and thickness of the GOri, respectively. The transformation functions $f_{E,v,\rho }\left(H_{GR},V_{GR},T\right)$ were derived by the genetic programming proposed by Zhao et al. [36,37]:

(6) $$f_{E}\left(H_{GR},V_{GR},T\right)=1.11-1.22V_{GR}-0.134\left(\frac{T}{T_{0}}\right)+0.559V_{GR}\left(\frac{T}{T_{0}}\right)\;-5.5H_{GR}V_{GR}+38H_{GR}V_{GR}^{2}-20.6H_{GR}^{2}V_{GR}^{2}\;f_{v}\left(H_{GR},V_{GR},T\right)=1.01-1.43V_{GR}+0.165\left(\frac{T}{T_{0}}\right)-16.8H_{GR}V_{GR}\;-1.1H_{GR}V_{GR}\left(\frac{T}{T_{0}}\right)+16H_{GR}^{2}V_{GR}^{2}\;f_{\alpha }\left(V_{GR},T\right)=1.01-2.01V_{GR}^{2}-0.0131\left(\frac{T}{T_{0}}\right)$$

**Figure 2:**
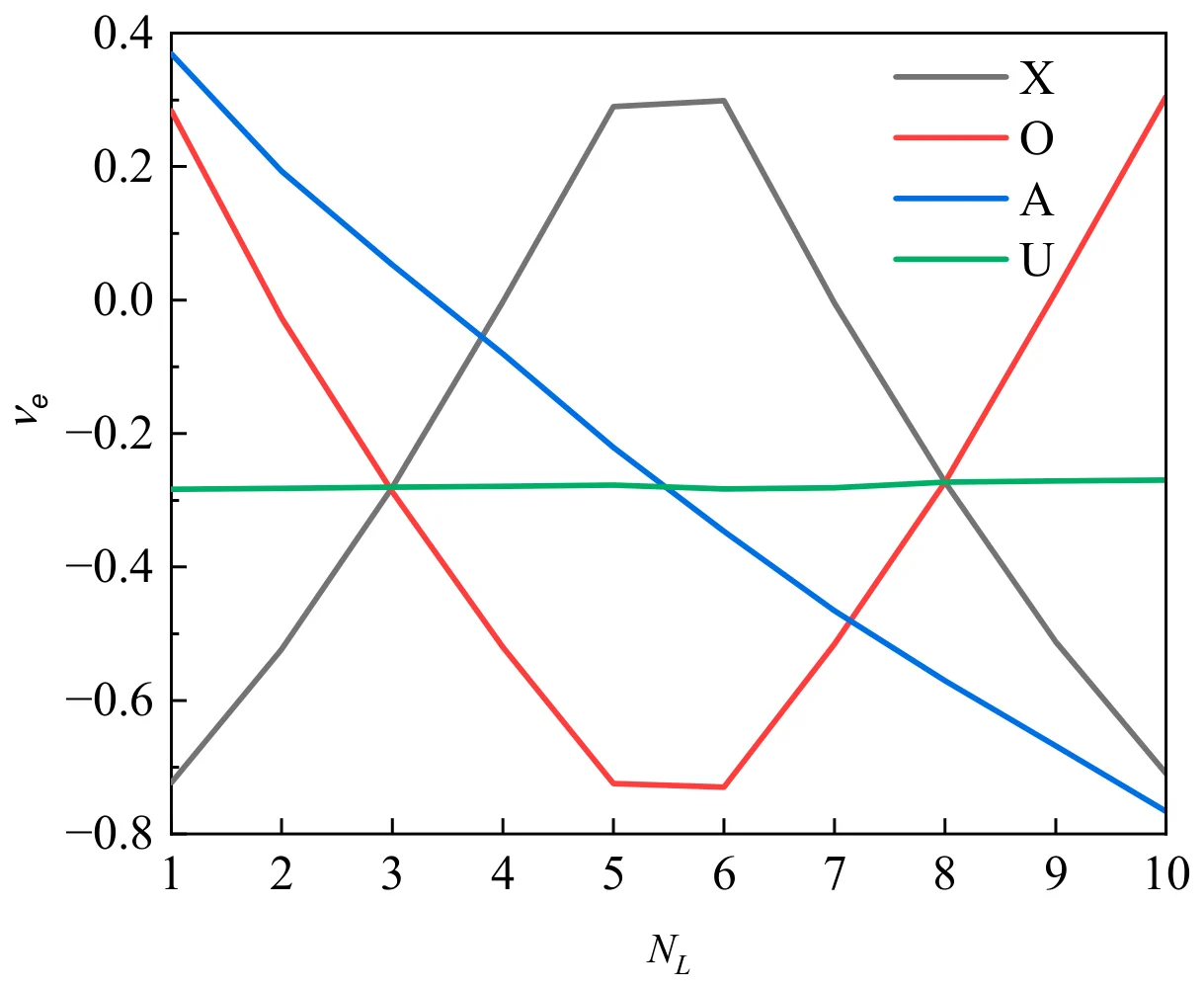
Effective Poisson’s ratio of the GOri-Cu composite beams as a function of the number of GOri layers (graphene mass fraction: 2.5 wt%, folding degree: 100%).

The temperature $T_{0}$ is set to 300 K, and the current ambient temperature is denoted as T. The distribution of the effective Poisson’s ratio along the thickness direction of the graphene origami-reinforced composite beams under different distribution conditions is shown in Figure 2. At a folding degree of 100%, the GOri-Cu composite structure may exhibit a negative Poisson’s ratio effect under all four volume distribution conditions. In the subsequent discussion, we will examine the buckling behavior of composite beams that exhibit a negative Poisson’s ratio effect as a function of the graphene origami content and degree of folding.

## 3. Fundamental Equations

### 3.1. Regular Equations

When the mechanical response of a GOri-Cu composite beam is studied from an energy perspective and when the composite beam is assumed to be linearly elastic, the Lagrangian function representing the energy of the GOri-Cu beam is given by the following equation:

(7) $$L=E_{K}-E_{P}=\frac{1}{2}I{\left(\frac{\partial \omega }{\partial t}\right)}^{2}-[\frac{1}{2}M_{x}\kappa +\frac{1}{2}N^{T}{\left(\frac{\partial w}{\partial x}\right)}^{2}+\frac{1}{2}M^{T}\kappa ]$$

In the equation, $E_{K}$ and $E_{P}$ represent the kinetic and potential energies of the system, respectively; $w\left(x,t\right)$ is the transverse deflection of the beam; t is time; κ is the curvature; I is the mass per unit length of the GOri-Cu composite beam; $\frac{1}{2}M_{x}\kappa =\frac{1}{2}D\kappa^{2}$ is the elastic bending strain energy, where D is the stiffness coefficient of the GOri-Cu composite beam; $N^{T}$ is the thermal axial force; and $M^{T}$ is the thermal bending moment. I, $D,N^{T}$ and $M^{T}$ are defined as follows:

(8a) $$I=b\int_{-h/2}^{h/2}\rho_{e}dz$$

(8b) $$D=b\int_{-h/2}^{h/2}E_{e}z^{2}dz$$

(8c) $$N^{T}=b\int_{-h/2}^{h/2}E_{e}\alpha_{e}\Delta Tdz$$

(8d) $$M^{T}=b\int_{-h/2}^{h/2}E_{e}z\alpha_{e}\Delta Tdz$$

In the equation, b is the beam width, and h is the beam thickness. Let w=q, $\kappa =-\frac{\partial^{2}w}{\partial x^{2}}$ and introduce the dual variable $p=\delta L/\delta \dot{q}=I\dot{q}$. Clearly, p is the momentum of the system, and δ represents the variational function. For any F, $\dot{F}=\frac{\partial F}{\partial t}$. From this, we obtain the Hamiltonian function as follows:

(9) $$H=p\dot{q}-L=\frac{p^{2}}{2I}+\frac{D}{2}{\left(\frac{\partial^{2}q}{\partial x^{2}}\right)}^{2}+\frac{1}{2}N^{T}{\left(\frac{\partial q}{\partial x}\right)}^{2}-\frac{1}{2}M^{T}\frac{\partial^{2}q}{\partial x^{2}}$$

In accordance with Hamilton’s principle, using the dual regular equations in the Hamiltonian framework, we obtain the following from the above equation:

(10) $$\dot{\psi }\equiv \left(\begin{array}{c} \dot{q}\\ \dot{p} \end{array}\right)=\left(\begin{array}{c} \frac{\delta H}{\delta p}\\ -\frac{\delta H}{\delta q} \end{array}\right)=\left(\begin{array}{c} \frac{p}{I}\\ -D\frac{\partial^{4}q}{\partial x^{4}}-N^{T}\frac{\partial^{2}q}{\partial x^{2}} \end{array}\right)$$

Consider a GOri-Cu beam fixed support. The first type of boundary condition, where the deflection and rotation are zero, is expressed in the Hamiltonian framework as follows:

(11) $$\left\{\begin{array}{c} q{|}_{x=0}=q{|}_{x=l}=0\\ \frac{\partial q}{\partial x}{|}_{x=0}=\frac{\partial q}{\partial x}{|}_{x=l}=0 \end{array}\right.$$

Moreover, considering the simply supported condition at both ends of the GOri-Cu composite beam, another set of boundary conditions-zero deflection and zero curvature-can be expressed in the Hamiltonian framework as follows:

(12) $$\left\{\begin{array}{c} q\left|x=0=q\left|x=l=0\right.\right.\\ \frac{\partial^{2}q}{\partial x^{2}}\left|x=0\right.=\frac{\partial^{2}q}{\partial x^{2}}\left|x=l\right.=0 \end{array}\right.$$

Unlike the first type of boundary condition, the simply supported constraint allows rotation at the beam ends, with the angle of rotation being unrestricted, whereas the fixed constraint completely restricts rotation. Equations (10)–(12) correspond, respectively, to the dual regularization equations and boundary conditions for the buckling problem of a GOri-Cu composite beam in symplectic space under the Hamiltonian framework.

### 3.2. Solving Regular Equations

First, a dimensionless transformation is performed by setting the following equation:

$$\xi =x/l,\;Q=w/l,\;\theta =N^{T}l^{2}/D$$

Here, θ is the critical buckling eigenvalue. The zero-order eigenvalue solution of the dual regularity equation satisfies $\dot{\psi }=0$, yielding the dimensionless form of the regularity equation:

(13) $$\frac{\partial^{4}Q}{\partial \xi^{4}}+\theta \frac{\partial^{2}Q}{\partial \xi^{2}}=0$$

The homogeneous linear Equation (13) has a general solution, as shown in Equation (14):

(14) $$Q=C_{1}+C_{2}\xi +C_{3}\cos(\sqrt{\theta }\xi )+C_{4}\sin(\sqrt{\theta }\xi )$$

(a) Based on the dimensionless boundary conditions fixed at both ends: $Q\left|\xi =0\right.=Q\left|\xi =1\right.=0$, $\partial_{\xi }Q\left|\xi =0\right.=\partial_{\xi }Q\left|\xi =1\right.=0$. The condition for Equation (12) to have a nonzero solution is as follows:

(15) $$\left[\begin{array}{cccc} 1 & 0 & 1 & 0\\ 0 & 1 & 0 & \sqrt{\theta }\\ 1 & 1 & \cos\sqrt{\theta } & \sin\sqrt{\theta }\\ 0 & 1 & -\theta \sin\sqrt{\theta } & \sqrt{\theta }\cos\sqrt{\theta } \end{array}\right]\left[\begin{array}{c} C_{1}\\ C_{2}\\ C_{3}\\ C_{4} \end{array}\right]=0$$

Expand and simplify to obtain the parameters θ that satisfy the following:

(16) $$2-2\cos\sqrt{\theta }-\sqrt{\theta }\sin\sqrt{\theta }=0$$

Using the normalization method, for the homogeneous linear system of Equation (13), let the integration constant be $C_{4}=1$, and solving yields the values of the remaining three constants:

(17) $$C_{1}=\frac{\sqrt{\theta }-\sin\sqrt{\theta }}{1-\cos\sqrt{\theta }},C_{2}=-\sqrt{\theta },C_{3}=\frac{\sin\sqrt{\theta }-\sqrt{\theta }}{1-\cos\sqrt{\theta }}$$

Substituting the above constants into Equation (14) yields the *n*-th buckling mode of the GOri-Cu composite beam:

(18) $$Q_{n}=\frac{\left(\sqrt{\theta_{n}}-\sin\sqrt{\theta_{n}}\right)}{\left(1-\cos\sqrt{\theta_{n}}\right)}[1-\cos(\sqrt{\theta_{n}}\xi )]+[\sin\left(\sqrt{\theta_{n}}\xi \right)-\sqrt{\theta_{n}}\xi ]$$

(b) Based on the dimensionless simply supported boundary conditions at both ends: $Q\left|\xi =0\right.=Q\left|\xi =1\right.=0$, $\frac{\partial^{2}Q}{\partial \xi^{2}}\left|\xi =0\right.=\frac{\partial^{2}Q}{\partial \xi^{2}}\left|\xi =1\right.=0$. The conditions for Equation (14) to have nonzero solutions are as follows:

(19) $$\left[\begin{array}{cccc} 1 & 0 & 1 & 0\\ 1 & 1 & \cos\sqrt{\theta } & \sin\sqrt{\theta }\\ 0 & 0 & \theta & 0\\ 0 & 0 & \theta \cos\sqrt{\theta } & \theta \sin\sqrt{\theta } \end{array}\right]\left[\begin{array}{c} C_{1}\\ C_{2}\\ C_{3}\\ C_{4} \end{array}\right]=0$$

Expanding this yields the condition θ such that the parameters satisfy the following:

(20) $$\theta^{2}\sin\sqrt{\theta }=0$$

By analogy with the above method, the nth buckling mode of the GOri-Cu composite beam in the corresponding case can be obtained as follows:

(21) $$Q_{n}=-\xi \sin\sqrt{\theta_{n}}+\sin(\sqrt{\theta_{n}}\xi )$$

## 4. Results and Discussion

### 4.1. Buckling Modeling

**Figure 3:**
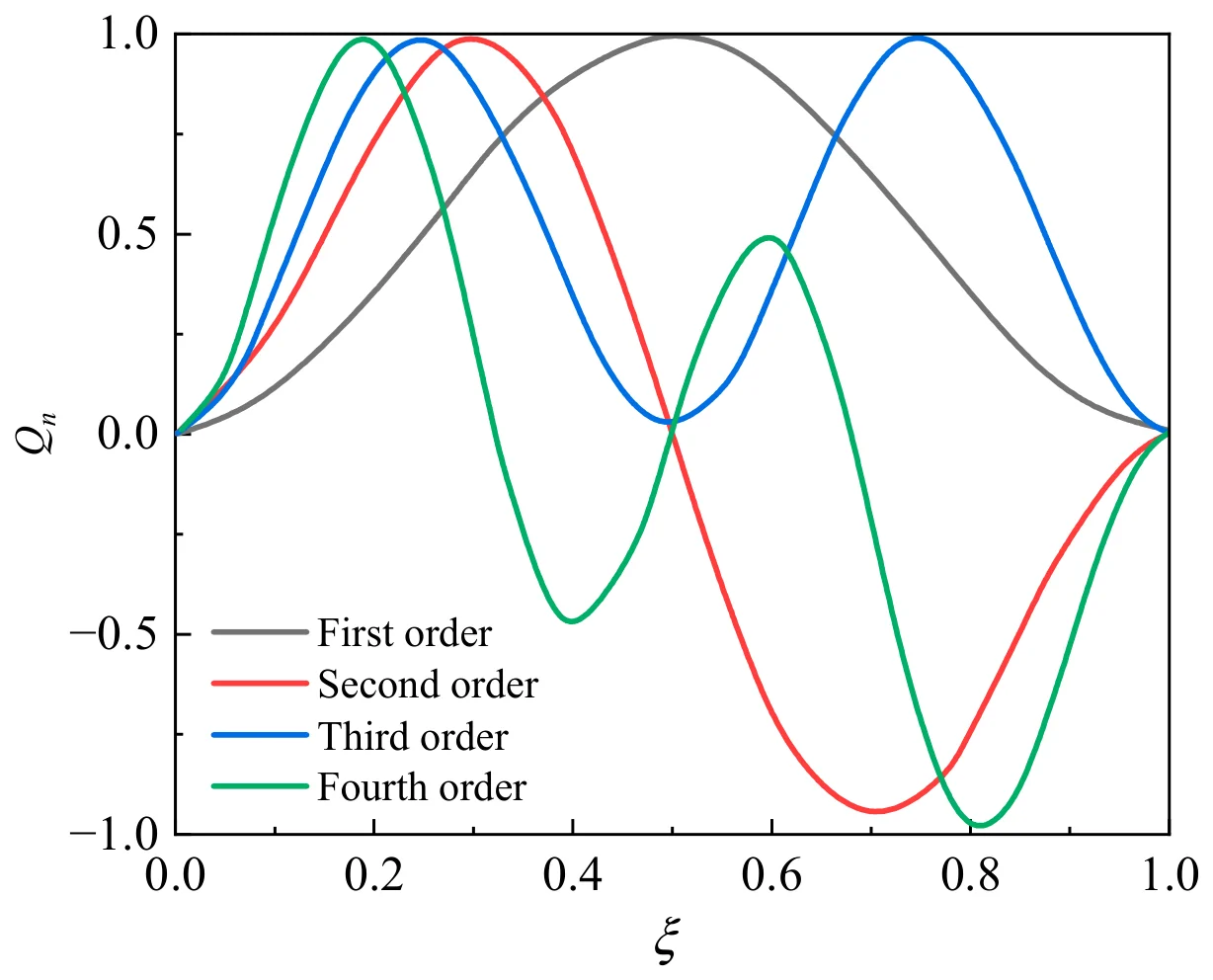
First- to fourth-order buckling modes for a fixed-end composite beam.

On the basis of Equation (14), which describes the buckling bifurcation conditions for the GOri-Cu composite beams under fixed boundary conditions, two sets of dimensionless eigenvalues can be obtained, with the corresponding values being $\theta_{1}=39.47$, $\theta_{2}=80.76$, $\theta_{3}=157.91$, $\theta_{4}=238.72$, $\theta_{5}=355.31$, $\theta_{6}=475.60$, $\theta_{7}=631.66$, $\theta_{8}=791.43$, $\theta_{9}=986.96$, and ⋯. Substituting the respective eigenvalues into the buckling mode, Equation (16) yields the buckling mode curves for each order. The buckling mode curves for orders 1 through 4 are shown in Figure 3.

**Figure 4:**
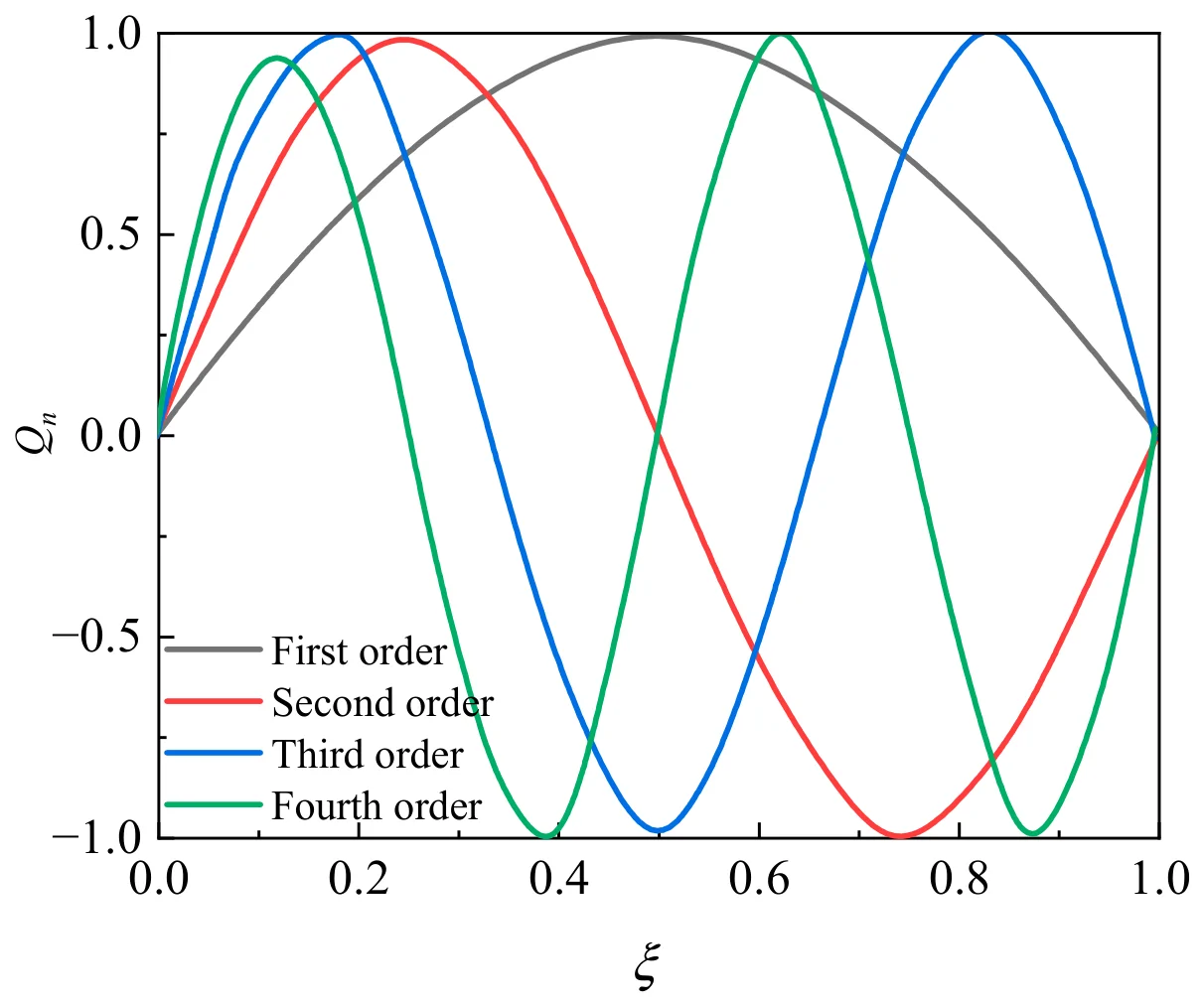
First- to fourth-order buckling modes for a simply supported composite beam.

Next, the other sets of dimensionless eigenvalues obtained by solving the bifurcation condition (18) under simply supported boundary conditions at both ends are $\theta_{1}=9.86$, $\theta_{2}=39.47$, $\theta_{3}=88.82$, $\theta_{4}=157.91$, $\theta_{5}=246.74$, $\theta_{6}=355.31$, $\theta_{7}=483.61$, $\theta_{8}=631.66$, $\theta_{9}=799.44$, and ⋯. Substituting these values into Equation (12) in turn yields the buckling modes of each order. The first four buckling modes of the GOri-Cu composite beam are shown in Figure 4.

### 4.2. Buckling Analysis

**Figure 5:**
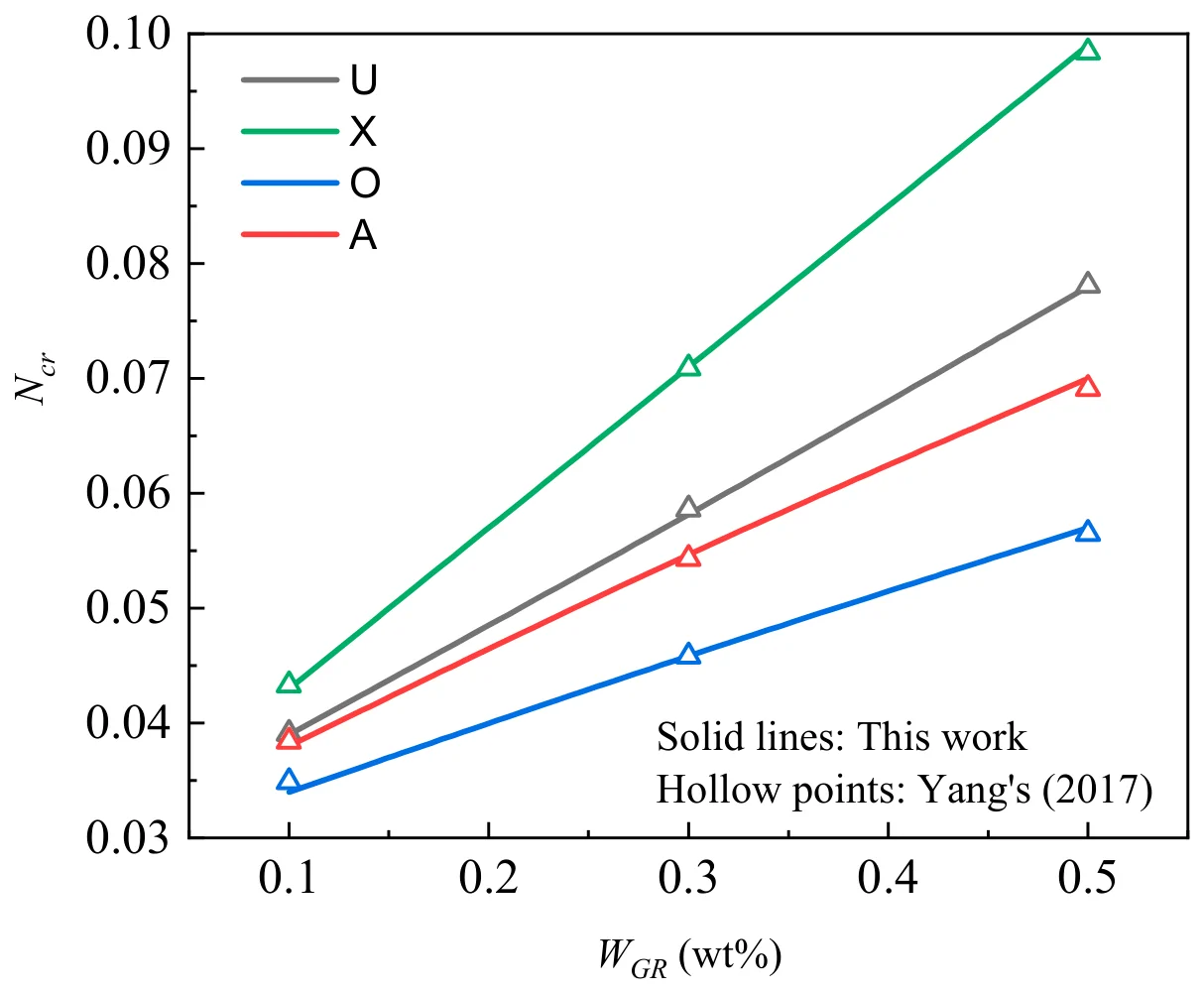
Comparison of the present results with those of Ref. [38] in terms of the variation in the dimensionless critical buckling load of a beam under C–C boundary conditions with changes in the graphene content.

First, to validate the proposed computational method, the mechanical behavior determined from the computational results was compared with established data in this field. To this end, we present the results obtained in this study side-by-side with those of Yang et al. [38] in Figure 5. This figure compares the dimensionless critical buckling thermal loads of composite beams with four different distribution patterns and varying mass fractions of graphene flakes. The patterns revealed by this comparison clearly demonstrate that the methodological framework developed in this study can accurately predict the buckling response of the beam structures under investigation.

To this end, we used the symplectic method to further investigate the influence and mechanisms of the degree of folding and graphene content of GOri on the buckling behavior of GOri-Cu composite beams under thermal loading for different volume fraction distribution patterns under fixed-end and simply supported boundary conditions. For the two boundary conditions—fixed and simply supported beams—let $N_{cr}=\frac{N^{T}l^{2}}{D_{0}}=\frac{\theta_{1}D}{D_{0}}$ and $D_{0}$ denote the flexural stiffness of pure copper, which $\theta_{1}$ has been determined using the symplectic method; it also represents the dimensionless critical buckling thermal load for a beam made of an isotropic, homogeneous material [39]. The following sections discuss the effects of $H_{GR}$ and $W_{GR}$ on the critical buckling load $N_{cr}$ under first-order buckling modes for fixed and simply supported conditions, respectively. The geometric parameters of the original graphene are $l_{Gr}=83.76$ Å and $t_{Gr}=3.4$ Å [37]. The material parameters at room temperature are shown in Table 1.

**Figure 6:**
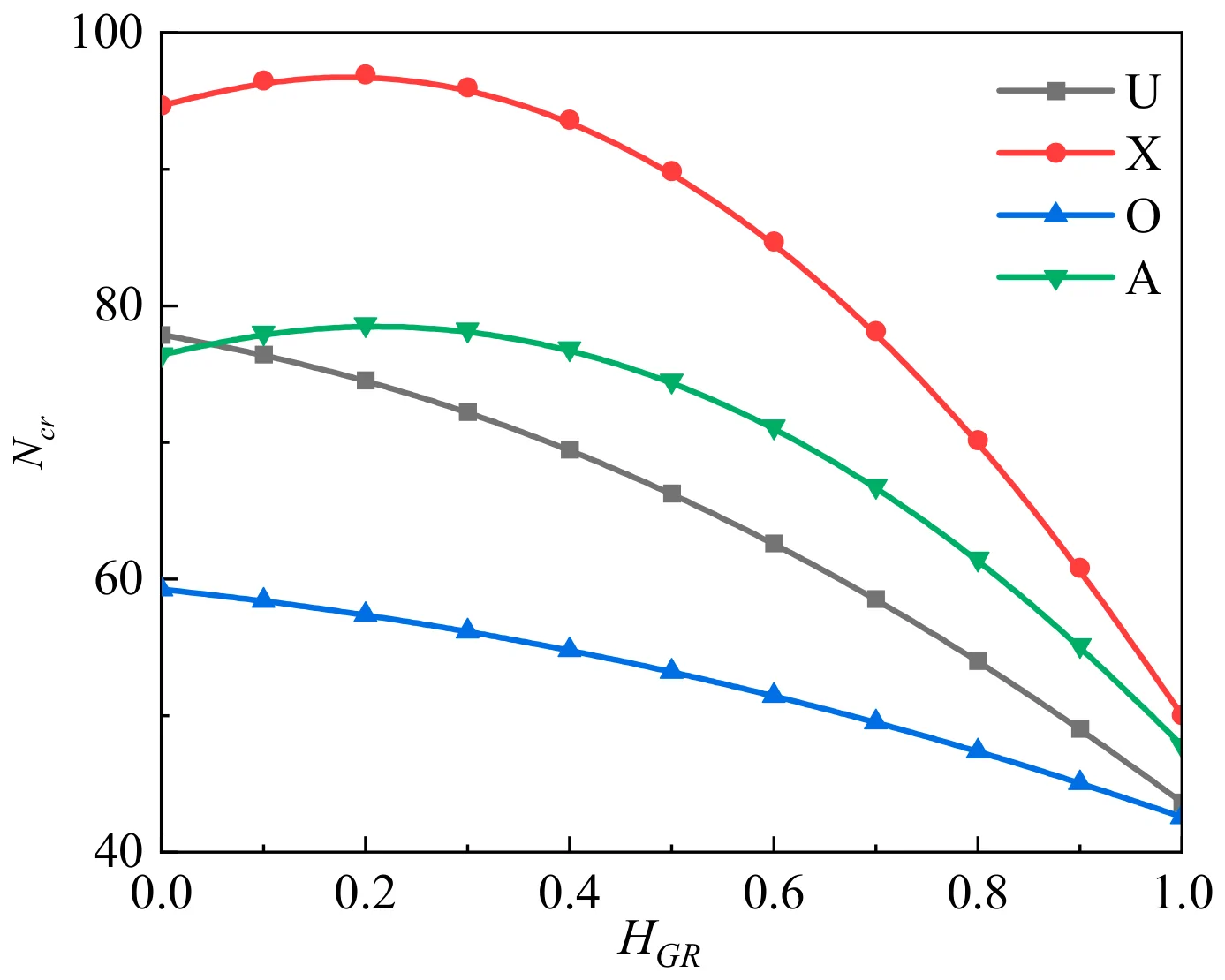
Relationships between the critical thermal load and degree of GOri folding under different volume fraction distribution patterns with fixed boundary conditions ($W_{GR}=2.5wt\%$).

**Figure 7:**
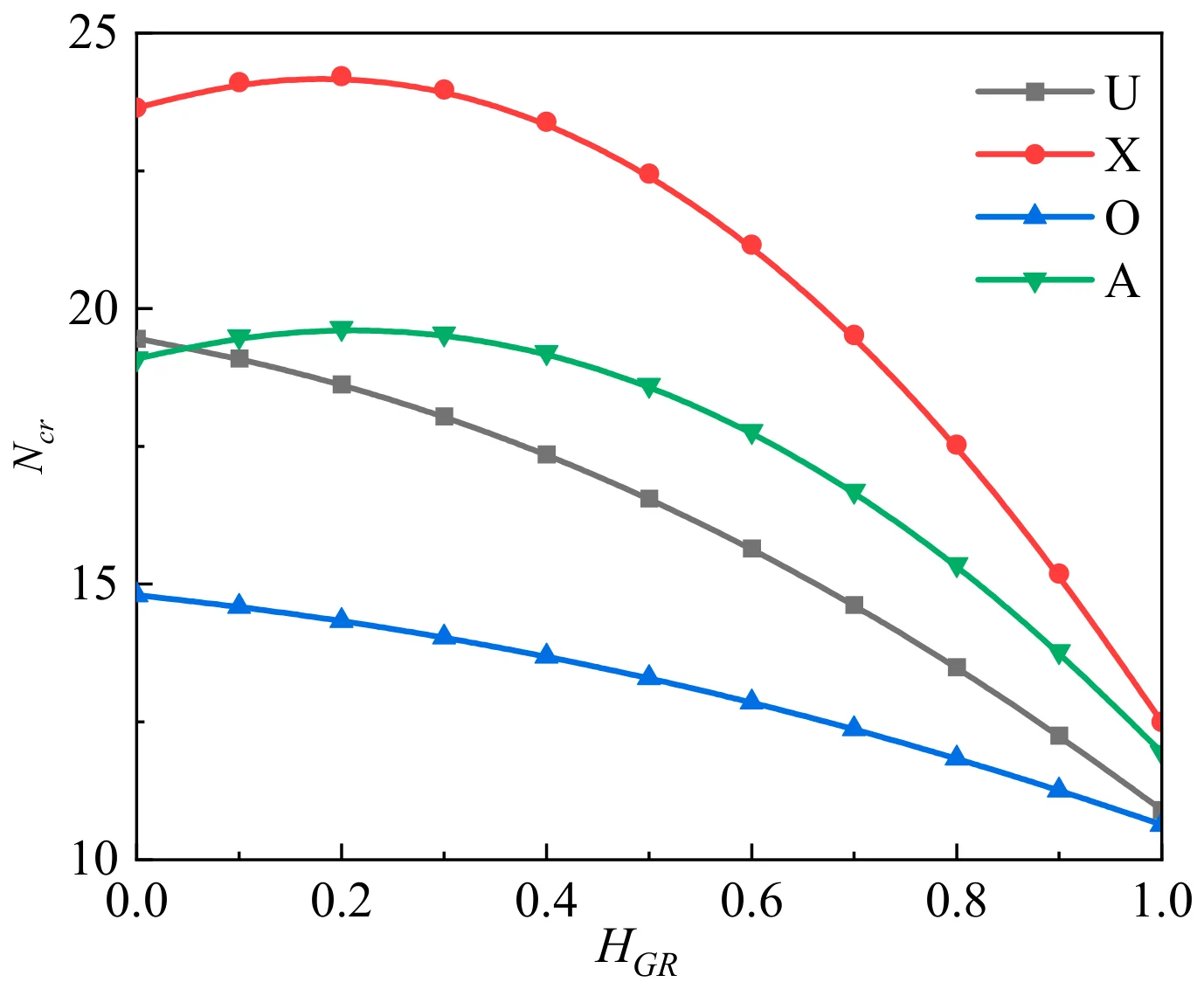
Relationships between the buckling thermal critical load and degree of folding of graphene origami under simply supported boundary conditions for different distribution patterns ($W_{GR}=2.5wt\%$).

As shown in Figure 6 and Figure 7, under both fixed and simply supported conditions, the buckling load $N_{cr}$ decreases as the degree of GOri $H_{GR}$ increases. This finding indicates that when the degree of GOri folding is small, the thermal load of the GOri-Cu composite beam increases to induce buckling; as the degree of folding increases, the critical buckling load decreases significantly. Therefore, increasing the folding degree of GOri reduces the buckling resistance of the functionally graded GOri-Cu composite structure, causing it to buckle under lower thermal loads. When $H_{GR}$ is constant, under the X-type distribution, the graphene origami is concentrated at the surface and sparse at the center; the resulting stress gradient provides greater resistance to buckling, whereas the O-type structure exhibits the poorest buckling resistance. Furthermore, when $H_{GR}$ is fixed, under the same volume fraction distribution pattern, the critical load of the simply supported beam is significantly greater than that of the fixed beam, indicating that boundary conditions have a significant effect on the critical load.

**Figure 8:**
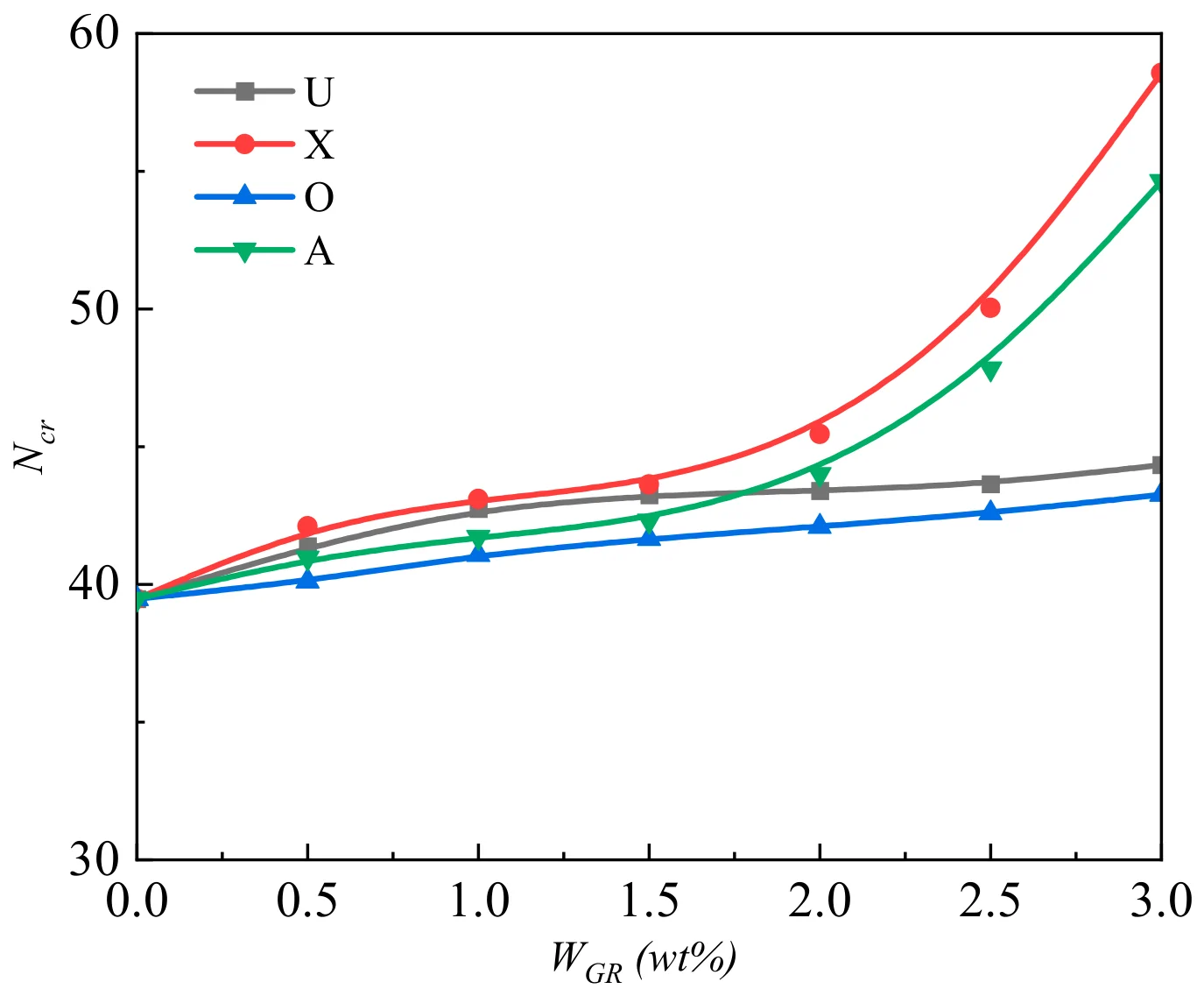
Relationships between the critical thermal load and graphene origami content under different distribution patterns with fixed boundary conditions ($H_{GR}=100\%$).

**Figure 9:**
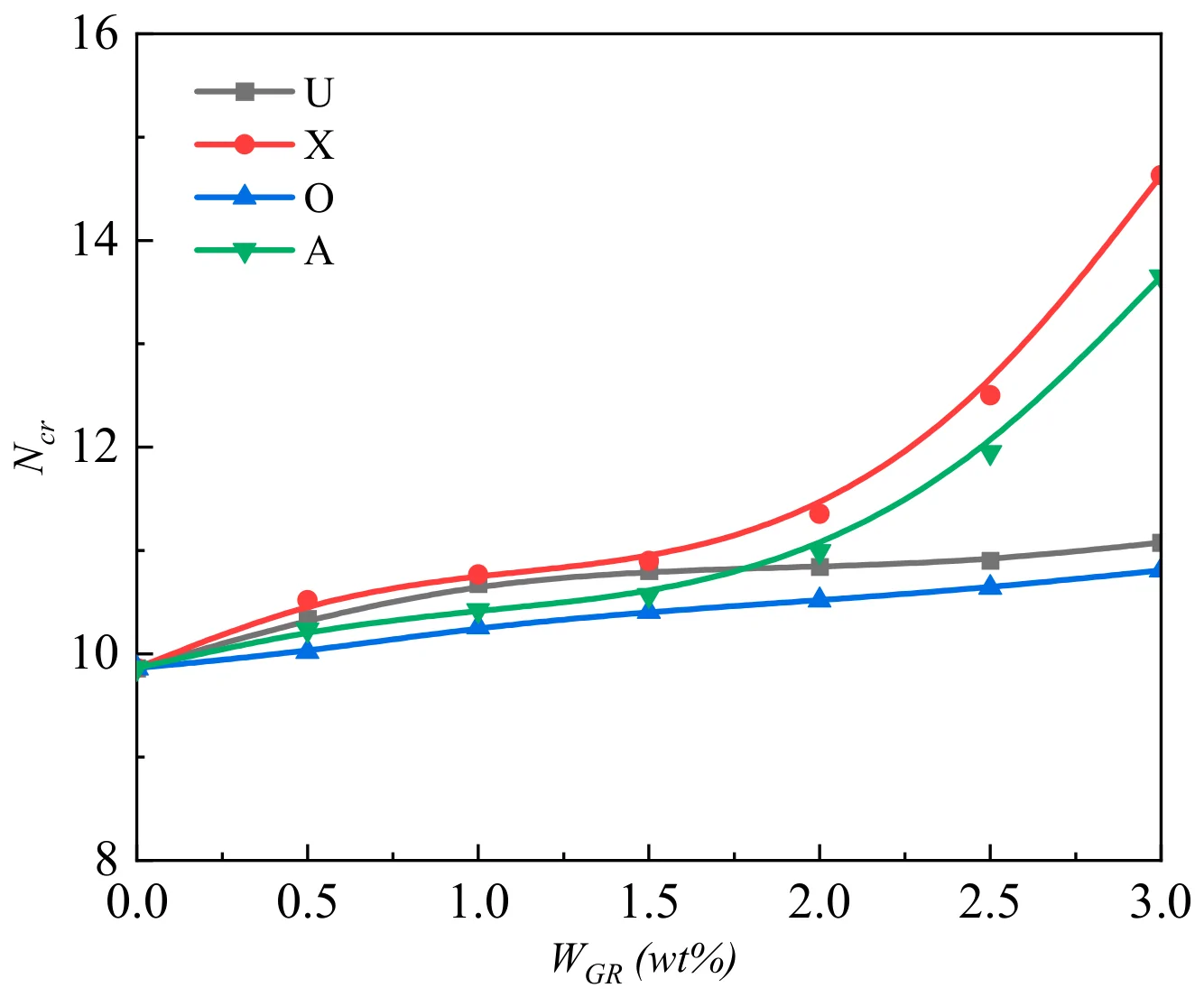
Relationships between the critical thermal load of buckling and the graphene origami content under different distribution patterns with simply supported boundary conditions ($H_{GR}=100\%$).

As shown in Figure 8 and Figure 9, under fixed and simply supported boundary conditions, the critical buckling load $N_{cr}$ increases with increasing $W_{GR}$. This indicates that a higher graphene content increases the critical buckling load, significantly enhances the elastic modulus of the composite material, and consequently improves the stiffness of the GOri-Cu beam, thereby enhancing the buckling resistance of the functionally graded GOri-Cu composite structure. Compared with other distribution patterns, the X-type distribution pattern provides better buckling resistance. When the volume fraction distribution pattern is fixed, the critical load of the GOri-Cu beam with both ends fixed is significantly greater than that of the simply supported GOri-Cu beam, further demonstrating that boundary conditions significantly affect the critical buckling load.

**Figure 10:**
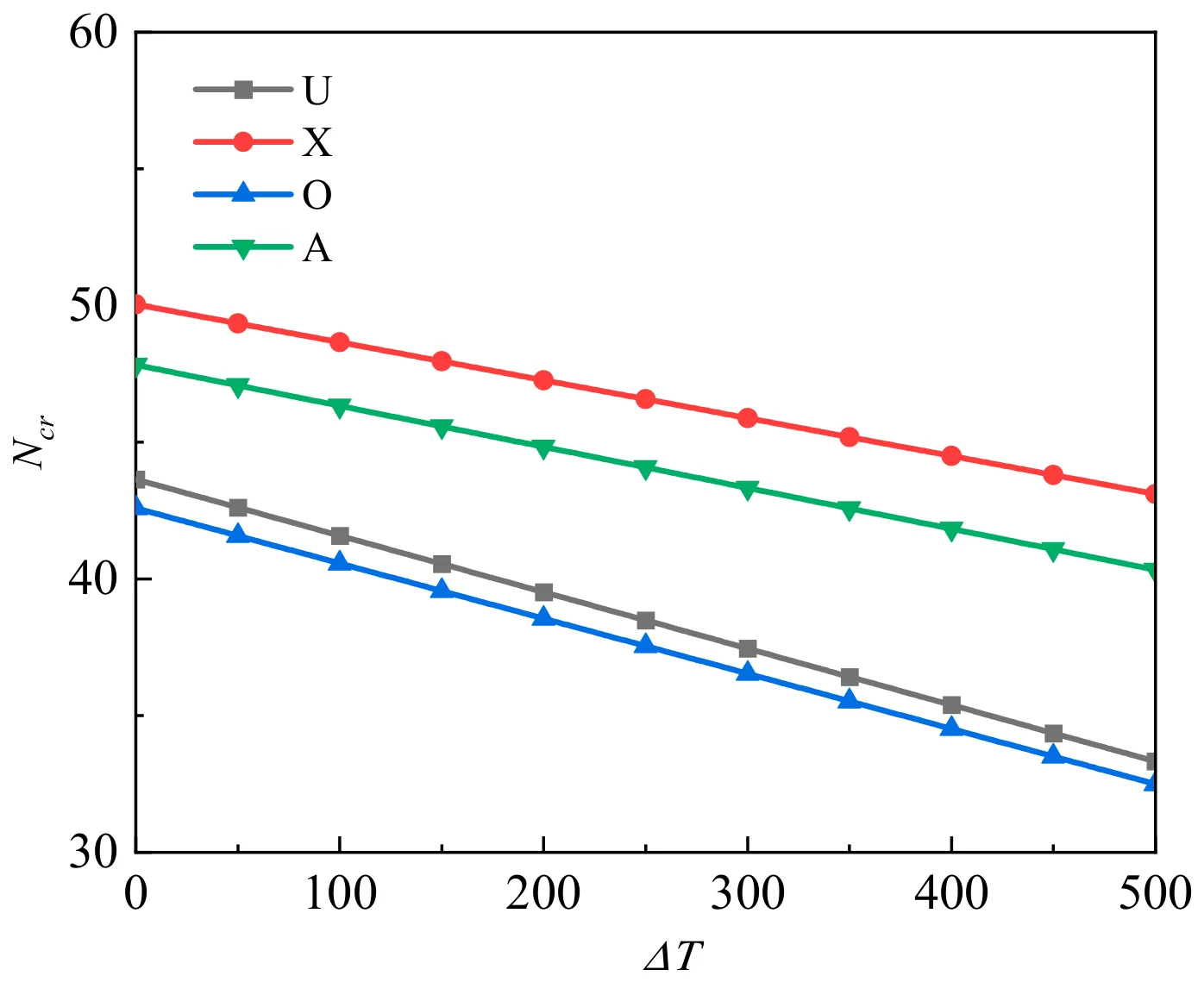
Variation in the critical buckling load versus temperature gradient for the four GOri distribution types.

The effect of material temperature on the critical thermal buckling load of simple supported beams of the GOri-Cu composite is shown in Figure 10. The results show that temperature has an inverse effect on the buckling load; that is, an increase in temperature leads to a decrease in the load value. Taking the X-shaped beam as an example, its critical load gradually decreases from approximately 50.04 at the reference temperature of 300 K to approximately 43.11 at 800 K, representing a decrease of 13.85%. When the analysis is extended to other beam types, the softening effect of temperature on the buckling resistance becomes even more pronounced. The buckling strengths of the A-shaped, U-shaped, and O-shaped beams decreased by 15.75% and 23.67%, respectively, and significantly increased by 23.95%. It can therefore be concluded that compared with other configurations, GOri-Cu composite beams with an X-shaped configuration exhibit lower sensitivity to temperature fluctuations in terms of their buckling resistance and demonstrate greater temperature stability.

## 5. Conclusions

On the basis of Euler–Bernoulli beam theory and by incorporating a Hamiltonian system to construct a buckling analysis model, this study conducted a systematic investigation into the stability of graphene origami composite beams. The results indicate that, within the framework of symplectic space, the buckling problem of graphene origami composite beams can be equivalently transformed into a zero-eigenvalue problem of the system, where the critical thermal load and buckling modes of the beam correspond to the symplectic eigenvalues and symplectic eigen solutions of the Hamiltonian system, respectively. Taking into account the continuity characteristics of graphene origami along the thickness direction and through the derivation of boundary conditions and normalization, this study successfully derived analytical solutions for the buckling modes and critical buckling load of the composite beam. The results indicate that the composite beam structure exhibits better buckling resistance when the graphene origami is distributed in the X- and A-patterns; as the graphene content increases, the critical buckling load of the composite beam increases accordingly. Furthermore, for the X- and A-pattern distributions, the critical buckling load of the composite beam first increases but then decreases when the degree of folding is considered, whereas both the U- and O-patterns tend to decrease; additionally, temperature has a weaker effect on the stability of the X-pattern structure. Furthermore, the variational framework established via Hamilton’s principle lays a solid foundation for subsequent research. On the basis of the present static thermal buckling analysis, future work can extend the symplectic elasticity approach to vibration characteristics, dynamic thermal buckling and post-buckling responses of graphene origami-reinforced composite structures. This will further enrich the application of symplectic analytical methods in the mechanical analysis of origami-based metamaterials.

## Figures and Tables

**Table 1:** Mechanical properties of graphene and Cu.

	Cu Matrix [37]	Graphene [37]
Young’s modulus (GPa)	69.75	929.57
Mass density (kg/m^3^)	8800	1800
Thermal expansion (1/K)	16.51 × 10^−6^	−3.98 × 10^−6^
Poisson’s ratio	0.38	0.22

## Data Availability

The original contributions presented in this study are included in the article. Further inquiries can be directed to the corresponding author.
